# Oral functional limitation and risk of frailty onset in older adults: a sex-stratified 4-Year cohort study with competing risk analysis

**DOI:** 10.1007/s11357-025-02015-8

**Published:** 2025-12-02

**Authors:** Kyung-Yi Do, Chang Won Won, Miji Kim, Joong-Yeon Lim

**Affiliations:** 1 Division of Population Health Research, Department of Precision Medicine, National Institute of Health, 200, Osongsaengmyeong2-Ro, Osong-eup, Heungdeok-gu, Cheongju-si, 28160 Chungcheongbuk-do Republic of Korea; 2 Elderly Frailty Sarcopenia Research Center, Department of Family Medicine, College of Medicine, Kyung Hee University Medical Center, Kyung Hee University, Seoul, Republic of Korea; 3 Department of Health Sciences and Technology, College of Medicine, Kyung Hee University, Seoul, Republic of Korea

**Keywords:** Oral function, Frailty, Chewing, Pronunciation, Cox model, Fine–Gray model, Older adults

## Abstract

**Background:**

Oral functional decline may contribute to frailty in older adults by affecting nutrition, physical performance, and psychosocial well-being. However, longitudinal evidence using rigorous analytic approaches, including competing risk analysis, remains limited. This study examined the association between self-reported oral functional limitation and frailty onset over 4 years, with attention to sex differences.

**Methods:**

We used 4-year prospective data from the Korean Frailty and Aging Cohort Study, comprising 1558 community-dwelling older adults aged 70–84 years at baseline (2016). After excluding participants with missing data and baseline frailty, 1348 robust individuals were analyzed. Oral function was assessed using two self-reported items on chewing and pronunciation difficulties. Frailty was defined using the validated Korean Frailty Index. Cox proportional hazards and Fine–Gray competing risk models were applied to evaluate the risk of frailty onset, stratified by sex. Interaction analyses were performed for major comorbidities.

**Results:**

Oral functional limitation was significantly associated with increased frailty risk (Cox-adjusted HR, 1.74; 95% CI, 1.20–2.53; Fine–Gray-adjusted SHR, 1.70; 95% CI, 1.17–2.46). This association was significant among women (HR, 2.44; 95% CI, 1.52–3.92; SHR, 2.36; 95% CI, 1.46–3.80), but not among men (HR, 0.87; 95% CI, 0.44–1.73; SHR, 0.86; 95% CI, 0.44–1.68). No significant interactions were observed with cardiovascular disease or osteoporosis.

**Conclusion:**

Self-reported oral functional limitation was significantly associated with frailty onset over 4 years, particularly among women. Incorporating oral function assessment into geriatric screening and community health programs may facilitate early identification of risk and the implementation of preventive strategies for frailty.

**Supplementary Information:**

The online version contains supplementary material available at https://doi.org/10.1007/s11357-025-02015-8.

## Introduction

In line with global trends, Korea has rapidly transitioned into a super-aged society (≥ 20% of the population aged ≥ 65 years) at the end of 2024, following a sharp increase in the elderly population [1]. This transition has contributed to increasing socioeconomic burdens related to caregiving and healthcare. Concurrently, the rising prevalence of frailty continues to threaten healthy aging [1, 2]

Frailty reflects biological aging and is associated with functional decline and loss of independence in older adults, contributing to reduced healthy life expectancy [3, 4]. Frailty is a geriatric syndrome characterized by multidimensional declines in physiological reserves, which increase vulnerability to adverse health outcomes such as falls, hospitalization, disability, impaired quality of life, and mortality [3, 5].

The concept of “oral frailty” has recently been introduced as a multidimensional construct describing the progressive decline of oral functions—particularly chewing, swallowing, and articulation—that reflect both physical and psychosocial aspects of aging [6]. Among these domains, chewing and articulation are considered core functional components linking oral health to overall frailty and are therefore the primary focus of the present study.

Oral functions are vital for maintaining nutrition, communication, and social engagement, all essential for healthy aging and the prevention of frailty in older adults [7]. Oral functional limitation can lead to dietary restriction, physical inactivity, and psychological distress, which in turn heighten vulnerability to frailty through behavioral pathways [8–11]. Older adults who report chewing discomfort have been shown to have a higher prevalence of chronic conditions and more than double the risk of frailty compared to those without such discomfort [10].

Furthermore, in a prospective Japanese community cohort (*n* = 2011), oral frailty (impairment in ≥ 3 of six oral measures) was associated with higher risks of incident physical frailty, sarcopenia, disability, and all-cause mortality (approximately 2.2–2.4 across outcomes) [12]. Additionally, a systematic review of 39 observational studies synthesized 12 oral indicators into four domains and reported consistent associations between poor oral health and frailty-related outcomes in community-dwelling older adults [13].

While prior studies have mainly focused on the onset of frailty, recent frameworks emphasize that frailty is not a fixed state but rather dynamic and potentially reversible. It may evolve along a continuum from robustness to prefrailty and frailty, and early intervention on modifiable risk factors can delay, and sometimes reverse, progression [5, 14, 15]. In this context, a decline in oral function, which affects both systemic and psychosocial health, has been proposed as an early marker and a modifiable contributor to frailty vulnerability [16–18]. Therefore, identifying older adults with oral functional limitations may enable timely preventive actions.

Although evidence on the association between oral health and frailty has grown [7, 13, 19], important gaps remain. First, many studies are cross-sectional, and operational definitions of oral frailty vary widely—from single symptoms to multi-domain composites—limiting comparability [7, 13, 20]. Second, few prospective cohorts have examined incident frailty; follow-up is often short, adjustment for key confounders is not systematic, and death during follow-up is rarely handled as a competing risk [7]. Because death prevents observation of later frailty onset, ignoring it can overestimate or bias risk.

Additionally, susceptibility to oral health deterioration may differ by sex and comorbidity status, although evidence on sex differences remains limited and inconsistent [21]. Postmenopausal hormonal changes have been suggested as one mechanism underlying women’s greater vulnerability to oral health decline [21–24].

Common age-related chronic conditions, including cardiovascular disease [25], diabetes [26], and osteoporosis [27, 28], are also linked to both oral health and frailty, but their potential modifying role in the oral health–frailty association remains uncertain.

Our objective was to evaluate whether subjective oral functional limitation is associated with incident frailty and whether this association differs by sex. We also examined potential modification by comorbidity status. We conducted a 4-year prospective analysis using data from the Korean Frailty and Aging Cohort Study (KFACS). We used sex-stratified Cox proportional hazards models and the Fine–Gray model, which treats death during follow-up as a competing event, to reduce bias and improve the accuracy of risk estimation.

## Methods

### Data source and participants

This prospective cohort study used data from the Korean Frailty and Aging Cohort Study (KFACS). KFACS is a nationwide, multicenter longitudinal study that recruited community-dwelling adults aged 70 to 84 years between 2016 and 2017 through quota sampling stratified by age (70–74, 75–79, 80–84 years) and sex across ten centers in Korea. This sampling method yielded 3014 participants, ensuring representation from both urban and rural populations nationwide [29].

To comprehensively assess participants’ physical, cognitive, social, and nutritional status, standardized tools were employed, including physical examinations (e.g., IPAQ), cognitive tests (e.g., MMSE), nutritional screening (e.g., MNA-SF), social and oral health assessments, and laboratory tests. Functional status was evaluated using activities of daily living (ADL) and instrumental activities of daily living (IADL). The cohort also included standardized biomarkers obtained from blood and urine tests (e.g., total cholesterol, triglycerides, HbA1c, and hs-CRP). The dataset encompasses self-reported health status, lifestyle factors, social relationships, and support networks, as well as objective physical and functional measures. The cohort was designed for biennial follow-up over 10 years to monitor changes in frailty and related health outcomes, such as disability, institutionalization, and mortality. Detailed information on the cohort design and data collection is available in the KFACS cohort profile [29].

This study uses data from the T1 (baseline, 2016–2017), T2 (2018–2019), and T3 (2020–2021) waves of the KFACS, in which participants were followed biennially. At the time of this study, only the 4-year follow-up data for the 2016 cohort (i.e., collected in 2020) had been finalized and made available in a cleaned format. In contrast, the corresponding 2021 data for participants enrolled in 2017 were not yet released in a harmonized format. Therefore, to ensure consistent follow-up duration and data completeness, we included only the 1558 participants recruited in 2016 who were followed up through 2020. Baseline characteristics are described for the full sample (*n* = 1558). Participants with missing frailty or oral function data, as well as those with baseline frailty, were excluded, yielding a final analytic sample of 1348 for incident frailty analyses. The detailed exclusion process is illustrated in Supplementary Fig. 1.

To estimate the risk of incident frailty, both Cox proportional hazards models (Cox-PH) and Fine–Gray competing risk models (Fine–Gray model) were employed. In the Cox model, participants who did not develop frailty, were lost to follow-up, or died were treated as censored. In the Fine–Gray model, death was treated as a competing event. Supplementary Fig. 1 presents the flow of participants included in the main analysis.

### Measurements

#### Frailty

Frailty was assessed using the Korean Frailty Index (KFI), an 8-item screening tool developed in 2010 by the Korean Geriatrics Society to provide a brief and feasible measure of frailty in Korean clinical and community settings [30]. The eight items were selected through expert consensus to capture multiple dimensions of frailty considered most relevant to older adults in Korea: (1) hospitalization history, (2) self-rated health (general health status), (3) polypharmacy (medication use), (4) unintentional weight loss (nutrition), (5) depressed mood (emotional state), (6) incontinence (functional status), (7) timed up-and-go test (mobility), and (8) hearing or vision impairment (communication ability) [30, 31]. Each item is scored 0 or 1, yielding a total score of 0 to 8. Based on previous studies, frailty status was classified as robust (0–2), pre-frail (3–4), and frail (≥ 5) [31, 32]. The detailed definitions, scoring rules, and questionnaire items for each component are provided in Supplementary Table 4.

The KFI was originally validated against the Cardiovascular Health Study (CHS) frailty phenotype, demonstrating acceptable sensitivity and specificity in identifying frailty [30]. Subsequent validation studies have further supported its criterion, construct, and predictive validity across various populations, including community-dwelling older adults in a nationwide cohort [32], population-based analyses using the National Health Insurance Service (NHIS) claims database [33], and primary-care settings [34]. Collectively, these findings confirm the KFI’s reliability and applicability as a multi-domain frailty measure in Korean older adults.

For time-to-event analyses, including both Cox proportional hazards and Fine–Gray competing-risks models, a dichotomous approach was used: individuals with scores of 0–4 were classified as robust, and those with scores of 5 or higher were considered frail (event).

#### Oral function limitations

Oral functional limitation in this study was defined as the two oral health items assessed in KFACS: self-reported chewing and pronunciation difficulties. These variables directly capture functional aspects of oral performance. This operational definition follows the framework of the Korea National Health and Nutrition Examination Survey (KNHANES) and has been applied in previous studies [35, 36]. The question regarding chewing difficulty was: “Are you currently experiencing discomfort when chewing food due to problems in your mouth, such as teeth, dentures, or gums?” The question regarding pronunciation difficulty was: “Do you experience difficulty in clearly pronouncing words due to issues with your teeth, dentures, gums, or other problems inside your mouth?” This combination aligns with international oral-frailty literature, which recognizes chewing and articulatory function as core domains of oral function [12, 19, 21], as supported by large-scale cohort evidence showing that both functions are major determinants of disability and mortality among older adults [37].

Each item was initially measured on a 5-point Likert scale (“very comfortable to very uncomfortable”), with higher scores indicating greater difficulty. For analysis, responses were recorded into a 0–4 scale, and the scores from both items were summed to yield a total score ranging from 0 to 8, with higher scores reflecting more pronounced oral functional limitation. This total score was used to conduct sensitivity analyses with a continuous scale, in addition to the dichotomized variable.

For categorization, each recorded item was classified as either “comfort” (including responses of “very comfortable,” “comfortable,” or “moderate”) or “discomfort” (including “uncomfortable” or “very uncomfortable”). Participants who reported “discomfort” on at least one of the two items were classified as having oral functional limitations. In contrast, those who selected “comfort” on both items were considered to have normal oral function.”

#### General characteristics and potential confounders

Demographic variables included age, sex, education duration (years), spouse status (absence or presence), and household income (categorized as high [≥ 5,000,000 KRW/month], middle [2,000,000 ≤ 5,000,000 KRW], or low [< 2,000,000 KRW or none]). Health-related factors included lifetime alcohol consumption experience (yes or no) and smoking experience, categorized as smoker (any lifetime smoking) or non-smoker (never smoked). Physical activity was assessed using the 7-day short-form self-report questionnaire from the International Physical Activity Questionnaire (IPAQ) and reported as the number of days per week engaging in moderate physical activity. Other health behaviors included self-reported sleep duration (hours per day and night), self-reported frequency of daily toothbrushing (times/day), and self-reported regular dental check-ups (yes/no). Nutritional status was measured using the Mini-Nutritional Assessment Short Form (MNA-SF), with total scores ranging from 0 to 14; higher scores indicate better nutritional status. Body mass index (BMI, kg/m^2^) was calculated and treated as a continuous variable. Cognitive function was evaluated using the Mini-Mental State Examination (MMSE; 0–30 points), with lower scores indicating poorer cognitive function.

Chronic health conditions included physician-diagnosed diabetes, dyslipidemia, osteoporosis, thyroid disease, and cardiovascular diseases. Cardiovascular disease burden (CVD_burden) was treated as a continuous variable indicating the number of coexisting cardiovascular conditions, including hypertension, myocardial infarction, angina pectoris (i.e., coronary artery disease), congestive heart failure, cerebrovascular disease (including stroke, cerebral infarction, and hemorrhage), and peripheral artery disease. Liver disease (0.8%) and chronic kidney disease (1.8%) were reviewed but excluded from the final models due to their low prevalence and potential to destabilize estimates. Polypharmacy was assessed using a self-reported item asking for the number of physician-prescribed medications taken for more than 3 months.

Laboratory biomarkers included serum total cholesterol (TC, mg/dL), triglycerides (TG, mg/dL), glycated hemoglobin (HbA1c, %), and high-sensitivity C-reactive protein (hs-CRP, mg/L).

### Statistical analysis

Statistical analyses were conducted using data from the Korean Frailty and Aging Cohort Study (KFACS), which followed community-dwelling adults aged 70–84 years for 4 years. Descriptive statistics were used to summarize baseline characteristics, with categorical variables presented as frequencies (%) and continuous variables as means with standard deviations.

Frailty status was categorized into three groups: robust, pre-frail, and frail. Group comparisons by sex were performed using chi-square tests for categorical variables and one-way ANOVA for continuous variables. Oral function was assessed using two approaches: as a continuous score (range 0–8) and as a binary variable (“comfort” vs. “discomfort”). For the continuous variable, p for trend was calculated using the original 5-point scale for chewing and pronunciation difficulties.

To examine the association between oral functional limitation and the risk of incident frailty, we employed both Cox-PH and Fine–Gray models, excluding individuals with baseline frailty (KFI ≥ 5). These models were chosen to appropriately account for censoring (Cox PH) and competing risks (Fine–Gray model). Analyses were performed using R version 4.1.1 with the survival and cmprsk packages. Proportional hazards assumptions were examined using Schoenfeld residuals in the Cox models and visually inspected in the Fine–Gray models, with no indication of violation.

Time to frailty onset was the dependent variable, and oral functional limitation was the main exposure. In the Cox model, participants who did not develop frailty, were lost to follow-up, or died during the observation period were treated as censored. In the Fine–Gray model, death was treated as a competing event to estimate the sub-distribution hazard of frailty onset.

Results from Cox-PH models were reported as hazard ratios (HRs) with 95% confidence intervals (CIs), and those from Fine–Gray models as sub-distribution hazard ratios (SHRs) with 95% CIs. Cumulative incidence functions (CIFs) were calculated to visualize the incidence of frailty and the competing risk of death over time. The number of events (frailty onset and death) was summarized by exposure group and sex using descriptive statistics to provide context for the competing risk analysis.

Multicollinearity was assessed using variance inflation factors (VIFs) for all covariates included in the fully adjusted Cox and Fine–Gray models; all variables showed acceptable VIFs ranging from 1.012 to 1.821.

We conducted both crude (unadjusted) and fully adjusted models to examine the association between oral functional limitation and incident frailty. Covariates for the fully adjusted models were selected based on theoretical relevance and prior literature identifying key demographic, behavioral, and clinical risk factors for frailty and oral health, rather than solely on statistical significance in univariate analyses [3, 17, 37–39]. The fully adjusted models included all covariates (sex, age, educational years, household income, spouse status, alcohol, smoking, physical activity, sleep duration, dental check-up, toothbrushing, body mass index, nutrition status, cognitive function, CVD_burden, osteoporosis, diabetes, dyslipidemia, thyroid dysfunction, polypharmacy, TC, TG, HbA1c, and hs-CRP). Household income was entered as an ordinal variable (higher codes indicating lower income) to evaluate a linear trend across categories. A complete list of covariates included in the fully adjusted models is provided in Supplementary Table 3*.*

Sensitivity analyses were conducted to assess the robustness of the main findings. In these analyses, oral function was treated as a continuous variable (0–8), and Fine–Gray models were applied using the same set of covariates as in the main analysis.

Interaction effect analysis was conducted by including a cross-product term (oral function limitation × comorbidity) in both Cox-PH and Fine–Gray models to assess whether the association between oral function limitation and frailty onset was modified by comorbidity status, specifically cardiovascular disease or osteoporosis.

All statistical analyses were performed using SAS version 9.4 (SAS Institute, Cary, NC, USA) and R version 4.1.1 (R Foundation for Statistical Computing, Vienna, Austria), including the survival, cmprsk, and forestplot packages. A two-sided p-value < 0.05 was considered statistically significant.

## Results

### General characteristics of participants at baseline (2016)

The study included 1558 community-dwelling older adults aged 70–84 years, with a mean age of 76.2 years (SD = 3.9); 47.0% were men and 53.0% were women. The average years of education were 7.95 (SD = 5.02), and 71.4% reported low household income.

At baseline, the mean body mass index was 24.4 kg/m^2^ (SD = 3.02), and the average nutritional status score (MNA-SF) was 12.84 (SD = 1.50), corresponding to the normal range (12–14 points) but approaching the lower threshold. The mean physical activity frequency was 2.92 days per week (SD = 2.59), and 33.4% of participants had received a dental check-up within the past year. Frailty (KFI ≥ 5) was identified in 9.9% (*n* = 149) of participants at baseline. The mean MMSE score was 25.42 (SD = 3.39), which is slightly above the cutoff for cognitive impairment (24 points), suggesting that most participants maintained preserved cognitive function. Among the six cardiovascular conditions assessed (hypertension, myocardial infarction, angina pectoris, stroke, congestive heart failure, and peripheral artery disease), 61.9% of participants had at least one condition (CVD = Yes). The mean CVD burden, defined as the total number of these conditions, was 0.72 (SD = 0.65). Other prevalent conditions were diabetes mellitus (21.2%), dyslipidemia (29.6%), thyroid dysfunction (4.7%), and osteoporosis (16.3%). The mean number of prescribed medications was 3.62 (SD = 2.99). Detailed baseline characteristics are provided in Supplementary Table 1.

#### Sex-stratified general characteristics by frailty group

Among 1501 participants, 9.9% (*n* = 149) were classified as frail, with a higher prevalence in women (11.8%) than in men (7.8%). In both sexes, frailty prevalence increased with age; the proportion aged ≥ 80 years with frailty rose substantially (*p* < 0.001). Education years decreased, and low household income was more common in the frail group for both men and women (*p* < 0.001). Among men, the proportion without a spouse was higher in the frail group (*p* = 0.001), whereas in women the difference was not significant (*p* = 0.093).

Physical activity days were significantly lower in frail compared with robust participants (men, 2.91 vs. 1.80; women, 2.24 vs. 1.19 days per week; *p* = 0.003 for men and *p* < 0.001 for women). Toothbrushing frequency was also lower in the frail group (*p* = 0.003 for men; *p* = 0.014 for women). No significant differences were observed in alcohol consumption, smoking, or BMI between frailty groups.

Nutritional status (MNA-SF) was significantly lower in frail participants compared with robust participants (men, 13.1 vs. 12.2; women, 13.1 vs. 11.9; *p* < 0.001), corresponding to the normal range in men and the “at risk of malnutrition” range in frail women. Cognitive function (MMSE) was also lower in the frail group than in the robust group (men, 26.5 vs. 25.5; women, 25.8 vs. 23.3; *p* < 0.001), and the mean score of frail women approached the cognitive impairment threshold (< 24 points).

Cardiovascular disease prevalence increased with frailty (men, 54.5% vs. 76.9%; women, 59.1% vs. 78.6%; *p* < 0.001), and CVD burden showed a similar pattern (*p* < 0.001). Osteoporosis was significantly more common in frail compared with robust participants (men, 1.7% vs. 7.4%, *p* = 0.004; women, 20.3% vs. 44.2%, *p* < 0.001). Diabetes showed a similar trend (men, 20.0% vs. 34.5%, *p* = 0.005; women, 13.1% vs. 33.0%, *p* < 0.001). Polypharmacy, defined as the number of physician-prescribed medications taken for more than 3 months, increased markedly with frailty (men, 2.8 vs 6.3; women, 2.6 vs 5.5; *p* < 0.001).

Total cholesterol levels were lower in the frail group than in the robust group in both men (*p* = 0.003) and women (*p* < 0.001). HbA1c levels were higher among frail participants of both sexes (*p* < 0.001). No significant differences were observed in hs-CRP or thyroid dysfunction.

Among women, frailty was associated with higher triglyceride levels (*p* = 0.004) and greater prevalence of dyslipidemia (*p* = 0.016). Among men, frailty was associated with a higher rate of recent dental check-ups (*p* = 0.047). Full sex-specific results are presented in Table 1..

**Table 1.** Baseline characteristics stratified by frailty status and sex (*n* = 1501)

	Men (*N* = 707)				Women (*N* = 794)			
	Robust	Pre-frail	Frail		Robust	Pre-frail	Frail	
Variables	484 (68.5)	168 (23.7)	55 (7.8)	*p-value*	435 (54.8)	265 (33.4)	94 (11.8)	*p-value*
Age (Mean ± SD)	76.0 ± 3.9	77.2 ± 3.8	78.1 ± 3.8	< 0.001	75.1 ± 3.7	76.7 ± 3.9	78.0 ± 3.9	< 0.001
Age group				< 0.001				< 0.001
70–74	197 (40.7)	44 (26.2)	13 (23.6)		212 (48.7)	87 (32.8)	20 (21.3)	
75–79	185 (38.2)	70 (41.7)	15 (27.3)		156 (35.9)	101 (38.1)	33 (35.1)	
> 80	102 (21.1)	54 (32.1)	27 (49.1)		67 (15.4)	77 (29.1)	41 (43.6)	
Education years	10.6 ± 4.5	9.0 ± 4.9	8.6 ± 4.4	< 0.001	7.3 ± 4.6	5.3 ± 4.4	3.6 ± 3.6	< 0.001
Household income				< 0.001				< 0.001
High	122 (26.7)	25 (15.5)	5 (9.8)		75 (19.1)	15 (6.4)	1(1.3)	
Middle	80 (17.5)	23 (14.3)	5 (9.8)		34 (8.7)	8 (3.4)	7 (9.2)	
Low	252 (55.8)	113 (70.2)	41 (80.4)		284 (72.3)	210 (90.1)	68 (89.5)	
Spouse status(no)	45 (9.3)	24 (14.3)	14 (25.5)	0.001	232 (53.3)	161 (60.8)	58 (61.7)	0.093
Alcohol (yes)	442 (91.3)	149 (88.7)	48 (87.3)	0.437	233 (53.7)	163 (61.7)	47 (50.0)	0.054
Smoking (yes)	381 (79.0)	134 (79.8)	42 (76.4)	0.865	8 (1.8)	9 (3.4)	5 (5.3)	0.132
PA (days/week)	2.91 ± 2.75	2.34 ± 2.69	1.80 ± 2.52	0.003	2.24 ± 2.40	1.86 ± 2.51	1.19 ± 2.06	< 0.001
Sleep duration	6.4 ± 1.4	6.3 ± 1.5	6.2 ± 1.6	0.652	6.0 ± 1.4	5.7 ± 1.6	5.6 ± 1.9	0.019
Dental check-up(no)	302 (62.5)	114 (67.9)	43 (78.2)	0.047	281 (64.7)	192 (72.5)	69 (73.4)	0.056
TB (times/days)	2.4 ± 0.9	2.1 ± 0.8	2.1 ± 0.8	0.003	2.5 ± 0.7	2.41 ± 0.7	2.21 ± 0.7	0.014
BMI index (kg/m^2^)	24.02 ± 2.82	23.76 ± 2.94	23.62 ± 3.45	0.447	24.89 ± 2.87	24.89 ± 3.21	24.86 ± 3.21	0.997
Nutrition status (MNA-SF)	13.13 ± 1.17	12.57 ± 1.67	12.18 ± 2.21	< 0.001	13.11 ± 1.23	12.68 ± 1.43	11.87 ± 2.07	< 0.001
Cognitive function (MMSE score)	26.5 ± 2.9	25.33 ± 3.1	25.49 ± 3.0	< 0.001	25.75 ± 3.15	24.05 ± 3.51	23.26 ± 3.87	< 0.001
CVD (yes)	262 (54.5)	107 (67.7)	40 (76.9)	< 0.001	250 (59.1)	172 (68.8)	66 (78.6)	< 0.001
CVD_burden	0.63 ± 0.65	0.84 ± 0.69	1.10 ± 0.77	< 0.001	0.63 ± 0.58	0.84 ± 0.70	0.92 ± 0.61	< 0.001
Osteoporosis (yes)	8 (1.7)	10 (6.0)	4 (7.4)	0.004	87 (20.3)	90 (34.5)	38 (44.2)	< 0.001
Diabetes (yes)	97 (20.0)	50 (29.9)	19 (34.5)	0.005	57 (13.1)	63 (24.0)	31 (33.0)	< 0.001
Dyslipidemia (yes)	103(21.5)	37(22.0)	12(22.6)	0.976	153 (35.5)	102 (39.7)	46 (51.7)	0.016
TGD (yes)	11 (2.3)	5 (3.0)	2 (3.6)	0.762	30 (6.9)	16 (6.1)	8 (8.7)	0.703
Polypharmacy	2.76 ± 2.49	5.60 ± 3.37	6.25 ± 2.59	< 0.001	2.58 ± 2.68	4.35 ± 2.82	5.52 ± 2.47	< 0.001
KFI score (0–8)	1.13 ± 0.75	3.41 ± 0.49	5.45 ± 0.71	< 0.001	1.23 ± 0.75	3.37 ± 0.48	5.40 ± 0.64	< 0.001
OF score (0–8)	1.94 ± 2.27	2.98 ± 2.68	3.56 ± 2.67	< 0.001	2.16 ± 2.29	2.97 ± 2.52	4.11 ± 2.48	< 0.001
TC (mg/dL)	170.9 ± 35.7	161.9 ± 34.2	159.5 ± 30.9	0.003	183.9 ± 35.5	172.2 ± 35.6	178.9 ± 34.2	< 0.001
TG (mg/dL)	119.0 ± 66.4	114.3 ± 58.02	125.6 ± 62.8	0.503	125.2 ± 56.7	121.6 ± 54.6	144.8 ± 79.5	0.004
HbA1C (%)	6.0 ± 0.7	6.1 ± 0.9	5.9 ± 0.6	0.223	6.0 ± 0.7	6.1 ± 0.7	6.4 ± 1.3	< 0.001
HS-CRP (m/L)	1.4 ± 2.1	1.6 ± 2.5	1.6 ± 1.9	0.498	1.2 ± 1.8	1.3 ± 2.0	1.0 ± 0.9	0.384

Note: values are presented as mean ± standard deviation or number (%). Group comparisons were performed using one-way ANOVA for continuous variables and chi-square test (or Fisher’s exact test) for categorical variables. CVD burden was calculated as the total number of six cardiovascular conditions: hypertension, myocardial infarction, congestive heart failure, angina pectoris, stroke, and peripheral artery disease. Polypharmacy refers to the number of medications currently taken. The OF score ranges from 0 to 8 and is the sum of two 5-point Likert scale items assessing subjective limitations in chewing and pronunciation (each scored 0–4; higher scores indicate greater limitation)

Abbreviations*: PA* physical activity, *TB* toothbrushing, *BMI* body mass index, *MNA-SF* mini nutritional assessment-short form, *MMSE* mini-mental status examination, *CVD* cardiovascular disease, *TGD* thyroid dysfunction, *KFI* Korean frailty index, *OF* oral function, *TC* total cholesterol, *TG* triglyceride, *HbA1C* glycated hemoglobin, *hs-CRP* high-sensitivity c-reactive protein

#### Association between oral functional limitation and frailty by sex

Among the 1497 participants, mean oral function scores increased progressively across frailty categories in both men (2.04 → 2.97 → 3.91, *p* < 0.001) and women (2.16 → 2.90 → 4.11, *p* < 0.001), indicating that poorer oral function was consistently associated with greater frailty.

When oral function was categorized into two groups, the prevalence of frailty was about two times higher in the discomfort group than in the comfort group (men, 11.7% vs. 5.4%; women, 17.9% vs. 6.6%; *p* < 0.001).

Ordinal analyses also showed a significant increasing trend in frailty prevalence with higher levels of chewing and pronunciation difficulty (p for trend < 0.001). For instance, among women who reported “very uncomfortable” chewing, 29.1% were frail, compared with only 5.0% of those who reported “very comfortable.”

These findings clearly show that declining oral function is strongly associated with higher frailty prevalence in both sexes. Full results are presented in Table 2..

**Table 2:** Association between oral function and frailty status by sex (Total *n*=1497)

	Man (*n*=706)				Women (*n*=791)			
Variables	Robust	Pre-frail	Frail	*p-value*	Robust	Pre-frail	Frail	*p-value*
OF* score	2.04 ± 2.28	2.97 ± 2.58	3.91 ± 2.55	< 0.001	2.16 ± 2.29	2.9 ± 2.52	4.11 ± 2.48	< 0.001
				*p*- trend				*p*- trend
OF group				< 0.001				< 0.001
Comfort	325 (73.7)	92 (20.9)	24 (5.4)		276 (64.6)	123 (28.8)	28 (6.6)	
Discomfort	158 (59.6)	76 (28.7)	31 (11.7)		158 (43.4)	141 (38.7)	65 (17.9)	
Specific items of OF								
Chewing difficulty				< 0.001				< 0.001
Very comfortable	235 (76.1)	60 (19.4)	14 (4.5)		184 (65.2)	84 (29.8)	14 (5.0)	
Comfortable	62 (68.9)	21 (23.3)	7 (7.8)		55 (55.6)	32 (32.3)	12 (12.1)	
Moderate	40 (69.0)	13 (22.4)	5 (8.6)		46 (70.8)	14 (21.5)	5 (7.7)	
Uncomfortable	118 (63.4)	49 (26.3)	19 (10.2)		117 (48.0)	94 (38.5)	33 (13.5)	
Very uncomfortable	29 (45.3)	25 (39.1)	10 (15.6)		32 (31.1)	41 (39.8)	30 (29.1)	
pronunciation difficulty				< 0.001				< 0.001
Very comfortable	335 (77.4)	78 (18.0)	20 (4.6)		287 (62.3)	140 (30.4)	34 (7.4)	
Comfortable	45 (51.7)	31 (35.6)	11 (12.6)		55 (48.7)	43 (38.1)	15 (13.3)	
Moderate	34 (66.7)	11 (21.6)	6 (11.8)		31 (52.5)	18 (30.5)	10 (16.9)	
Uncomfortable	62 (54.4)	39 (34.2)	13 (11.4)		55 (41.4)	51 (38.3)	27 (20.3)	
Very uncomfortable	7 (33.3)	9 (42.9)	5 (23.8)		7 (26.9)	12 (46.2)	7 (26.9)	

*P*-values obtained using ANOVA and chi-square tests. P for trend calculated using linear-by-linear association test (Cochran–Armitage trend test). *OF* Oral Function

#### Cumulative incidence of frailty by oral function (Fine–Gray model)

Over the 4-year follow-up, the cumulative incidence of frailty was higher in the oral functional discomfort group (15.0%, 80/533) than in the comfort group (8.5%, 69/815).

The difference was more pronounced in women (20.4%) than in men (8.1%), as shown in Supplementary Table 2.

**Fig. 1:**
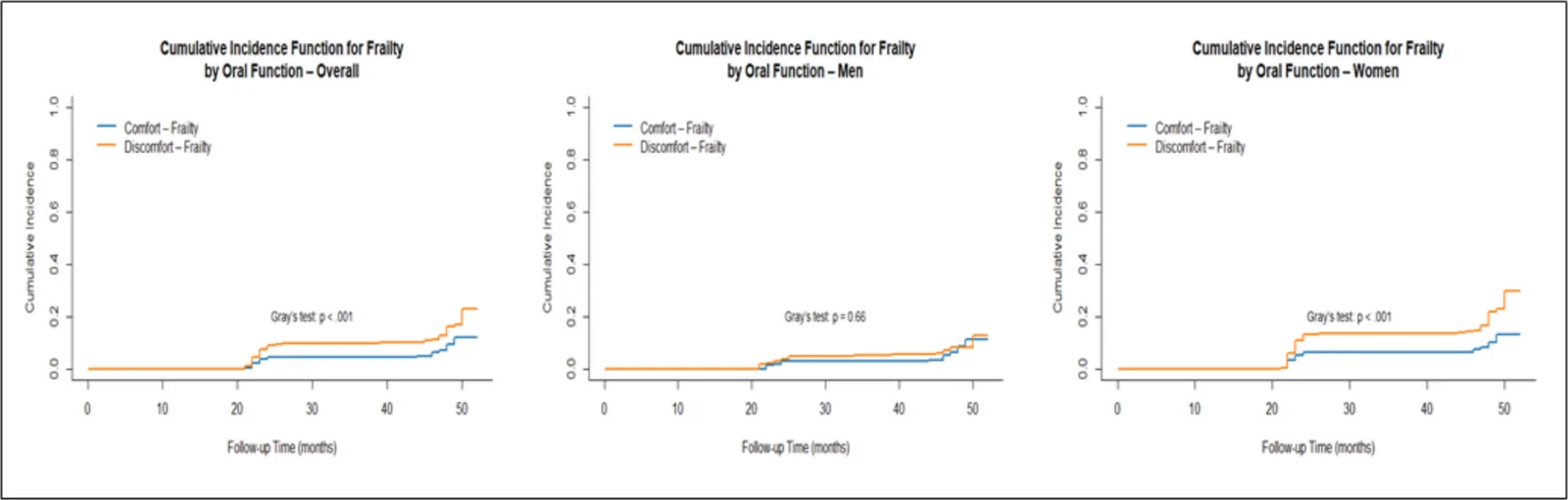
Cumulative incidence function (CIF) for frailty by oral functional status. Fine–Gray competing risk model curves are shown for the overall sample, men, and women. Gray’s test was used to compare incidence differences between oral functional groups (comfort vs. discomfort)

Cumulative incidence function (CIF) curves demonstrated a consistently higher incidence of frailty in the discomfort group (Gray’s test *p* < 0.001). In sex-stratified analyses, this difference remained significant in women (*p* < 0.001) but not in men (*p* = 0.66), highlighting a clear sex-specific pattern (Fig. 1).

#### Impact of oral functional limitation on the risk of incident frailty: cox-PH and fine–gray models

**Fig. 2:**
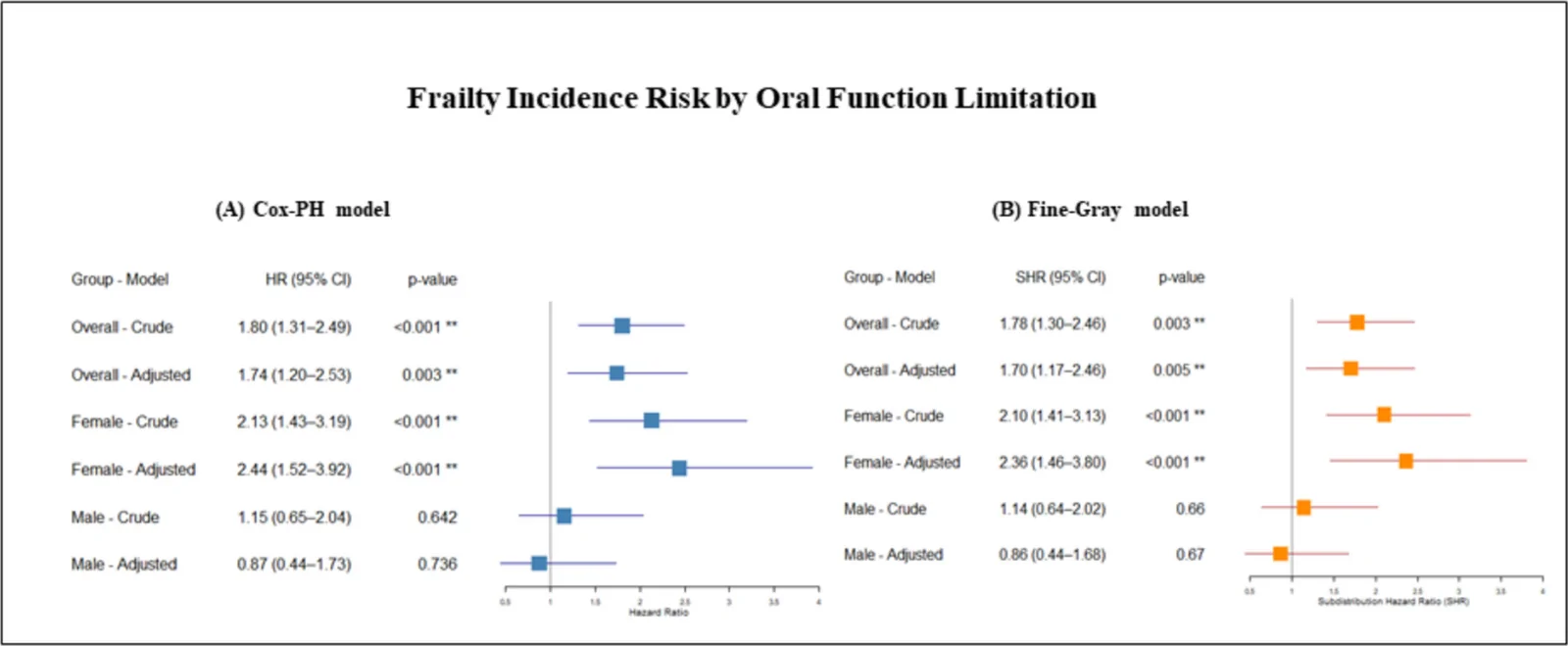
Association between oral functional limitation and incident frailty over 4-years. (A) Cox proportional hazards models. (B) Fine–Gray competing risk models. Results are shown for crude and fully adjusted models, stratified by sex. HR: Hazard Ratio; SHR: Sub-distribution Hazard Ratio; CI: Confidence Interval

In the fully multivariable models adjusting for all covariates, oral functional limitation was significantly associated with an increased risk of incident frailty in both the Cox PH model (HR 1.74, 95% CI 1.20–2.53) and the Fine–Gray model (SHR 1.70, 95% CI 1.17–2.46). In sex-stratified analyses, this association remained significant only in women (Cox HR 2.44 [1.52–3.92]; Fine–Gray SHR 2.36 [1.46–3.80]) but not in men (HR 0.87 [0.44–1.73]; SHR 0.86 [0.44–1.68]). These results were consistent across both modeling approaches and adjustment levels, confirming the robustness of the observed association between oral functional limitation and frailty onset (Fig. 2).

Sensitivity analyses treating oral function as a continuous score in the Fine–Gray competing risk model yielded results consistent with the main findings (overall, Fine–Gray SHR 1.08 [1.01–1.16]), further supporting the robustness of the associations. As in the main analysis, significant associations were observed in women (Fine–Gray SHR 1.17 [1.08–1.28]), but not in men (Fine–Gray SHR 0.90 [0.78–1.03]) (Supplementary Fig. 2).

#### Interaction effects of oral functional limitation with comorbidities on frailty risk: cox-PH and fine–gray models

Interaction analyses were conducted to determine whether the association between oral functional limitation and frailty onset differed by baseline comorbidities, specifically cardiovascular disease (CVD) and osteoporosis. Interaction terms between oral function and each comorbidity were incorporated into both Cox proportional hazards and Fine–Gray competing risk models.

**Fig. 3:**
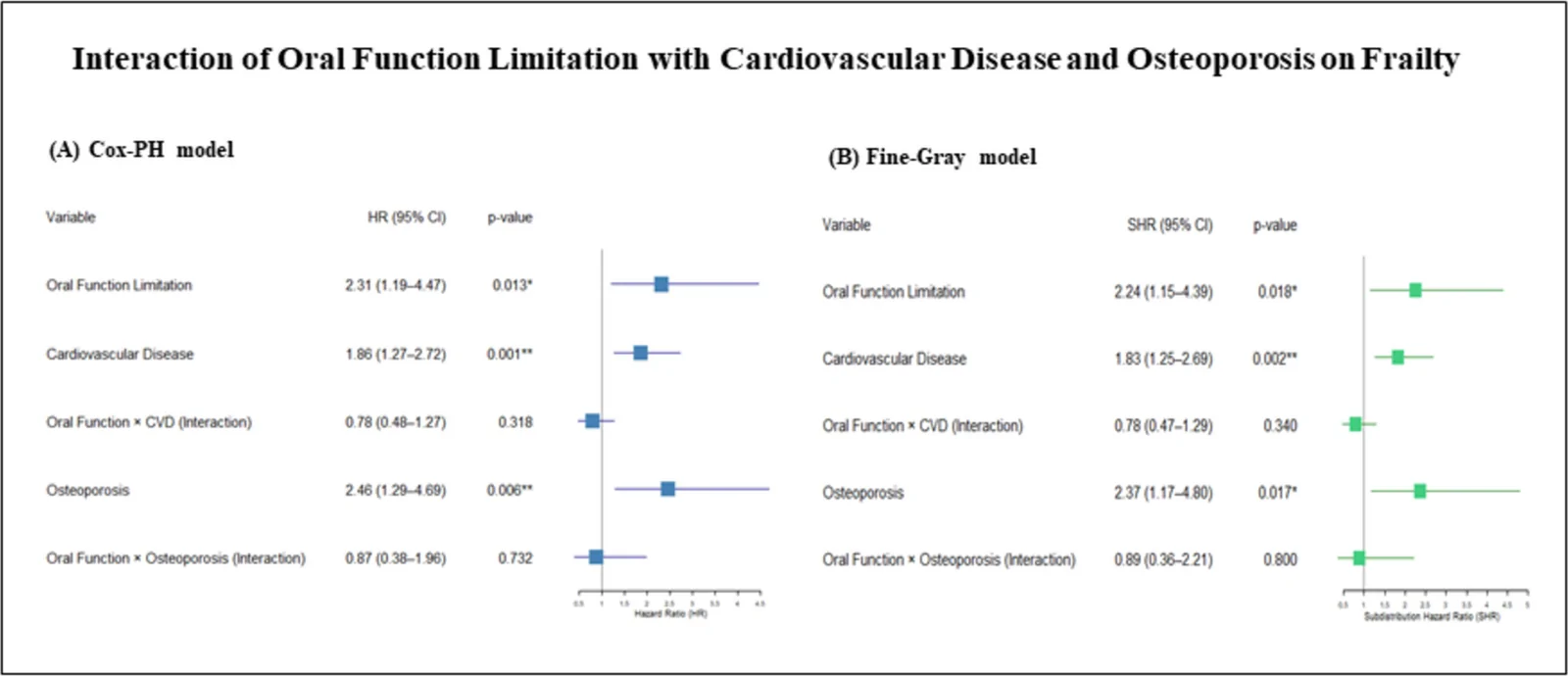
Interaction effects of oral function limitation and comorbidities on frailty risk. Interaction terms tested: oral function × cardiovascular disease; oral function × osteoporosis. Fully adjusted models used the same covariates as in the main analysis. HR (95% CI): Hazard Ratio (95% confidence interval); SHR: Sub-distribution Hazard Ratio

Oral functional limitation remained significantly associated with a higher risk of frailty in both models (Cox HR = 2.31 [95% CI 1.19–4.47]; Fine–Gray SHR = 2.24 [1.15–4.39]). Both cardiovascular disease (CVD) and osteoporosis were also associated with increased frailty risk. However, no significant interaction effects were found for oral function × CVD (Cox HR = 0.78 [0.48–1.27]; Fine–Gray SHR = 0.78 [0.47–1.29]) or oral function × osteoporosis (Cox HR = 0.87 [0.38–1.96]; Fine–Gray SHR = 0.89 [0.36–2.21]), indicating no evidence of effect modification (Fig. 3).

## Discussion

In this prospective cohort study, self-reported oral functional limitation was significantly associated with an increased risk of incident frailty among community-dwelling older adults. In sex-stratified analyses, the association was significant only among women, who had nearly twice the risk of developing frailty, whereas no association was observed in men. These findings underscore the importance of early assessment and intervention targeting oral function as a potential strategy for frailty prevention, particularly among older women. The use of the Fine–Gray competing risk model further strengthened methodological rigor by accounting for death as a competing event, a factor of particular relevance in aging cohorts. The persistence of the association between oral functional limitation and frailty onset in this model supports the robustness and clinical relevance of our results.

Previous studies from Japan, Taiwan, and Europe have consistently reported that impaired masticatory function is associated with an increased risk of frailty and mortality, underscoring its importance for healthy aging [40–43]. Both objective measures (e.g., bite force, chewing efficiency) and subjective assessments have been used to evaluate masticatory function in these studies [10, 39, 42, 44, 45]. A recent meta-analysis confirmed that self-reported chewing difficulty is significantly associated with frailty, supporting the validity of subjective assessments in large-scale epidemiologic research [46]. Notably, several studies have also reported a higher prevalence of oral health problems and frailty among older women [16, 17, 43, 47], a pattern consistent with our findings.

Several plausible mechanisms may underlie the relationship between oral function and frailty. First, chewing and pronunciation difficulties represent distinct but interrelated aspects of oral functional decline, often resulting from tooth loss, oral pain, or mucosal disease. Both are key domains in the Japanese concept of oral frailty, along with swallowing dysfunction, oral dryness, poor hygiene, and tooth loss [12, 37]. Although they share common etiologic origins, their pathways to frailty may differ. Impaired mastication can lead to nutritional imbalance and reduced protein and micronutrient intake, contributing to sarcopenia and functional decline, thereby increasing frailty risk [10, 44, 48].

Women may be particularly vulnerable due to sex-specific characteristics such as lower baseline muscle mass, postmenopausal hormonal changes, and reduced dietary protein intake [49]. Although the association between oral functional limitation and frailty onset was not statistically significant in men, this finding should be interpreted with caution.

In our study, the prevalence and severity of chewing and pronunciation difficulties, as well as the occurrence of frailty during follow-up, were substantially lower in men than in women. This imbalance likely led to fewer frailty events and insufficient statistical power to detect a significant association. Therefore, the nonsignificant result for men probably reflects limited power rather than the absence of a true relationship, highlighting the need for confirmation in studies with larger male samples.

Second, pronunciation difficulties may restrict verbal communication and social engagement, leading to isolation, psychological distress, and depressive symptoms, well-known psychosocial determinants of frailty [50]. Previous evidence from a nationally representative Korean study reported that pronunciation problems had a greater adverse impact on health-related quality of life in older women than in men, suggesting that women may be more sensitive to psychosocial distress and communication difficulties related to oral function [51]. In line with these findings, recent systematic reviews have shown that poor oral health is associated with loneliness and social isolation [52], reinforcing the psychosocial pathway linking oral function to frailty. While chewing difficulty primarily reflects a physical–nutritional mechanism, pronunciation difficulty acts through psychosocial and communicative pathways, together representing complementary aspects of oral function relevant to frailty.

Third, chronic oral inflammation (e.g., periodontitis) may induce systemic inflammation, promote muscle catabolism, and impair protein synthesis, leading to muscle loss and functional decline. Supporting this, Kimble et al. [18] reported that accumulated oral health problems were associated with increased frailty progression over time [18]. Furthermore, in women, postmenopausal estrogen deficiency may reduce salivary secretion and increase mucosal dryness and inflammation, amplifying oral discomfort and frailty susceptibility [23, 53].

Overall, these mechanisms suggest that the stronger association observed in women reflects both biological susceptibility and a greater oral health burden. National health statistics in Korea similarly show that older women are more likely than men to report chewing discomfort and to have fewer remaining teeth, reinforcing this sex-specific disparity [54]. An additional possibility is that cognitive function (MMSE) was lower in the frail group than in the robust group, and this difference was more pronounced among women (robust vs. frail MMSE, men 26.5 vs. 25.5; women 25.8 vs. 23.3; *p* < 0.001). Previous studies in Korea and other Asian populations have shown that lower cognitive function is closely associated with frailty [55] and that the prevalence of cognitive frailty is higher in women than in men [56]. These findings suggest that sex-related differences in cognition may partly help explain the stronger association between oral function and frailty observed in women in our study.

In this study, oral functional limitation was assessed using self-reported measures of chewing and pronunciation difficulties, which capture subjective impairments that are not readily detected by conventional clinical indicators. Compared with objective assessments that require specialized tools or trained personnel, self-reported evaluations are more practical for large-scale population studies and particularly valuable in geriatric screening settings [57], including both chewing and pronunciation domains, which allowed for a broader assessment of oral function, encompassing physical and psychosocial aspects relevant to frailty in older adults.

Furthermore, oral function comprises multiple domains, including chewing, pronunciation, swallowing, oral dryness, and the number of remaining teeth. Among these, difficulties in chewing and pronunciation have been consistently identified as key indicators reflecting oral functional decline [13, 37]. However, because the KFACS dataset included only these two items, the measure’s limited scope precluded formal psychometric validation, including internal consistency and test–retest reliability. Therefore, our findings should be interpreted with caution, acknowledging this measurement constraint and the operational definition used.

However, this approach also has limitations. It did not assess other important domains such as swallowing dysfunction or oral dryness, and the binary classification into “comfort” and “discomfort” may have oversimplified the continuum of oral functional decline. To address this, we conducted sensitivity analyses using the original Likert-scale items and a composite continuous score (range 0–8), which yielded consistent results across models. These analyses support the reliability of self-reported oral function measures and highlight the need for future studies to include objective, multidimensional assessments of oral health in relation to frailty.

Additionally, it is important to consider the strengths and limitations of the main outcome measure used in this study, the Korean Frailty Index (KFI). Although the KFI has demonstrated acceptable sensitivity and specificity in validation studies based on the CHS frailty phenotype [30], it remains a brief screening tool. It cannot fully capture the multidimensional domains assessed by a comprehensive geriatric assessment (CGA). While the KFI is supported by several validation studies [30–34] and is widely used in both community and clinical settings, its diagnostic performance relative to the full CGA remains limited. Therefore, findings based on the KFI should be interpreted with these constraints in mind, and future studies directly comparing the KFI with CGA-based assessments are warranted to further establish its diagnostic utility.

Furthermore, our competing risk analysis using Fine–Gray models confirmed that oral functional limitation was significantly associated with an increased risk of frailty onset, even after accounting for the competing risk of death. This underscores the robustness and clinical relevance of oral function as an early marker for frailty. In additional analyses, we tested interactions with cardiovascular disease and osteoporosis, but no significant effect modification was observed in either the Cox or Fine–Gray models. These findings suggest that the association between poor oral function and frailty is consistent regardless of comorbidity status.

From a clinical perspective, cardiovascular disease and osteoporosis are closely linked to both oral health and frailty through shared mechanisms involving systemic inflammation, vascular dysfunction, and nutritional compromise. The coexistence of chronic conditions such as hypertension, diabetes, and osteoporosis with persistent oral functional impairment may have additive or synergistic effects, potentially accelerating frailty progression [25, 58, 59]. Although no statistical interaction was observed in our analysis, these findings underscore the importance of oral health as a modifiable, preventive target, even among older adults without major chronic diseases.

### Study strengths

Its prospective, 4-year multicenter design provides robust evidence from a large community-based cohort of older adults in Korea, one of the few Asian populations with standardized longitudinal frailty assessments. The use of both Cox proportional hazards and Fine–Gray competing risk models enabled rigorous estimation of frailty risk while appropriately accounting for mortality as a competing event. Consistent findings across both categorical and continuous operationalizations of oral function further support the robustness of the results. Stratified analyses by sex and comorbidity status added depth, demonstrating that the associations remained stable across clinically relevant subgroups. Together, these methodological refinements and subgroup insights enhance the validity and generalizability of the findings, underscoring the clinical feasibility of incorporating simple oral function screening into frailty prevention strategies for community-dwelling older adults.

#### Study limitations

First, participants were recruited using quota sampling stratified by age and sex. This design reflects the population’s demographic distribution to some extent, but as it is non-random, it carries a risk of selection bias and limited generalizability. Moreover, relatively healthy older adults living in the community may have been more likely to participate, potentially underrepresenting less healthy individuals; therefore, survivor bias cannot be ruled out. Second, oral functional status was assessed using self-reported measures, which are subject to recall or perception bias. Nevertheless, this approach remains practical for large-scale geriatric assessments and was partially addressed through sensitivity analyses using a continuous composite score.

Third, although our models included a broad set of relevant covariates, certain systemic conditions, such as liver or kidney dysfunction, were excluded because of their very low prevalence and estimation instability. Their omission is unlikely to have materially influenced the observed associations. Fourth, because all variables were measured at baseline, time-varying changes in oral function or health status that could influence frailty risk over time were not examined. Additionally, psychosocial determinants, including social support and emotional well-being, were not captured and warrant further investigation. Future research incorporating broader psychosocial and environmental factors would help clarify their contribution to the oral function–frailty relationship. Although the 4-year follow-up period is substantial, longer-term studies are needed to better understand longitudinal pathways and evaluate the dynamic trajectory of frailty. Finally, as this study was based on community-dwelling older adults in Korea, the findings may not be fully generalizable to populations in other regions, given potential differences in ethnicity, culture, and healthcare systems.

These findings underscore the need to integrate oral health into geriatric care and frailty prevention strategies. In Korea, national health check-ups and community-based screening programs should include routine oral health assessments and standardized diagnostic tools for oral frailty, such as the number of remaining teeth, masticatory performance, swallowing function, oral dryness, and oral hygiene, similar to those implemented in Japan. Establishing an integrated system that links dental examinations, cohort-based data collection, and policy implementation would provide a stronger evidence base for oral health-oriented frailty prevention. Also, targeted support should be prioritized for socially and economically disadvantaged older adults, who face a higher burden of poor oral function and unmet dental care needs.

## Supplementary Information

ESM 1 (PNG 84.2 kb)

High resolution image (TIF 98.7 kb)

ESM 2 (PNG 151 kb)

High resolution image (TIF 75.2 kb)

ESM 3 (DOCX 17.5 kb)

ESM 4 (DOCX 37.7 kb)

## Data Availability

The KFACS dataset analyzed in this study is not publicly accessible due to institutional data protection regulations. Data cannot be shared at this time.
