# Supplementary material for: Oral functional limitation and risk of frailty onset in older adults: a sex-stratified 4-Year cohort study with competing risk analysis
Source: GeroScience. 2025 Dec 2;48(5):6801–15. doi: 10.1007/s11357-025-02015-8 (PMC13601412; doi:10.1007/s11357-025-02015-8)
Supplement: Supplementary file 1 [file 11357_2025_2015_MOESM3_ESM.docx]

Supplementary figure legends

**Supplementary Figure 1. Flowchart showing participant eligibility and final analytic sample (n = 1,348)**

**Supplementary figure 2.** **Sensitivity analysis of frailty incidence risk based on oral function score using Fine–Gray competing risk models.**

Forest plots present subdistribution hazard ratios (sHR) and 95% confidence intervals for the association between oral function score (0–8) and incident frailty, stratified by sex.

**Note:** Models adjusted for all covariates included in the main analysis.
