# Supplementary material for: Oral functional limitation and risk of frailty onset in older adults: a sex-stratified 4-Year cohort study with competing risk analysis
Source: GeroScience. 2025 Dec 2;48(5):6801–15. doi: 10.1007/s11357-025-02015-8 (PMC13601412; doi:10.1007/s11357-025-02015-8)
Supplement: Supplementary file 2 [file 11357_2025_2015_MOESM4_ESM.docx]

**Supplementary Tables (1-4)**

| Supplementary Table 1. General characteristics of study participants at baseline (2016) | | | |
| --- | --- | --- | --- |
| General characteristics total n=1558 | | | |
| Variables | Category | N (%) or Mean ± SD | |
| Sex | Male | | 733(47.0) |
|  | Female | | 825(53.0) |
| Age |  | | 76.23±3.93 |
| Age group | 70-74 | | 592(38.0) |
|  | 75-79 | | 678(43.5) |
|  | >80 | | 288(18.5) |
| Education status (years) |  | | 7.95±5.02 |
| Household income | High | | 244(17.2) |
|  | Middle | | 161(11.4) |
|  | Low | | 1012(71.4) |
| Spouse status | Absence | | 558(35.8) |
|  | Presence | | 999(64.2) |
| Alcohol experience | Yes | | 1123(72.2) |
| Smoking | Yes | | 600(38.6) |
| Physical activity | Days/week | | 2.29±2.59 |
| Sleep duration | Hour/ day(night) | | 6.08±1.50 |
| Dental check-up | Yes | | 519(33.4) |
| Toothbrushing | Times/day | | 2.35±0.79 |
| Body Mass Index (kg/m^2^) |  | | 24.40±3.02 |
| Nutritional status | MNA-SF score, 0-14 | | 12.84±1.50 |
| Cognitive function | MMSE Score, 0-30 | | 25.42±3.39 |
| Frailty | KFI ≥5 | | 149(9.9) |
| Korean Frailty Index score | (0-8) | | 2.24±1.59 |
| Oral Function score | (0-8) | | 2.52±2.49 |
| Diabetes | Yes | | 329(21.2) |
| Dyslipidemia | Yes | | 461(29.6) |
| Cardiovascular disease (CVD) | Yes | | 928(61.9) |
| CVD_burden | CVD_count (0-6) | | 0.72±0.65 |
| Osteoporosis | Yes | | 249(16.3) |
| Thyroid dysfunction | Yes | | 72(4.7) |
| Polypharmacy |  | | 3.62±2.99 |
| Total cholesterol (mg/dL) |  | | 173.97±35.83 |
| Triglyceride (mg/dL) |  | | 122.54±61.87 |
| HbA1C (%) |  | | 6.02±0.76 |
| hs-CRP (mg/L) |  | | 1.35±1.97 |
|  |  | |  |

Descriptive statistics are presented as mean ± standard deviation or number (%).

**Notes:** CVD_burden: Number of cardiovascular conditions (hypertension, myocardial infarction, angina pectoris, congestive heart failure, stroke, peripheral artery disease). Polypharmacy: Number of medications currently taken. Oral function score: Sum of chewing and pronunciation limitation scores (range 0–8).

**Abbreviations:** HbA1C, Glycates Hemoglobin; hs-CRP, High-sensitivity C-reactive protein.

|  | Supplementary Table 2. Four-year incidence of frailty by oral function status | | | | | | | | |  |
| --- | --- | --- | --- | --- | --- | --- | --- | --- | --- | --- |
|  | |  | | Incidence of frailty | | | | |  |  |
| Oral function status | | Total no. | Event n (%) | | Death n (%) | Censored n (%) | Follow-up time (Year) | Total person-years | | |
| All subjects | | 1348 | 149(11.0) | | 45(3.3) | 1154(85.6) | 3.9 [3.8-4.0] | 5046.9 | |  |
| Comfort | | 815 | 69(8.5) | | 26(3.2) | 720(88.3) | 3.9 [3.8-4.0] | 3082.9 | |  |
| Discomfort | | 533 | 80(15.0) | | 19(3.6) | 434(81.4) | 3.9 [3.8-4.1] | 1960.2 | |  |
| Man | | 650 | 49(7.5) | | 32(4.9) | 569(87.6) | 3.9 [3.8-4.0] | 2440.0 | |  |
| Comfort | | 416 | 30(7.2) | | 19(4.6) | 367(88.2) | 3.9 [3.8-4.0] | 1568.5 | |  |
| Discomfort | | 234 | 19(8.1) | | 13(5.6) | 202(86.3) | 3.9 [3.8-4.0] | 867.7 | |  |
| Woman | | 698 | 100(14.3) | | 13(1.9) | 585(83.8) | 3.9 [3.8-4.1] | 2606.9 | |  |
| Comfort | | 399 | 39(9.8) | | 7(1.8) | 353(88.5) | 3.9 [3.8-4.0] | 1514.4 | |  |
| Discomfort | | 299 | 61(20.4) | | 6(2.0) | 232(77.6) | 3.9 [3.8-4.1] | 1092.5 | |  |

**Note:** Cumulative incidence, death, censoring status, and total person-years are reported for each oral function group (comfort vs. discomfort), stratified by sex. Follow-up time is expressed as median [interquartile range, IQR].

Supplementary Table 3. Predictors of incident frailty from baseline characteristics (Fine–Gray model)

| **Variable** | **SHR (95% CI)** | **p-value** |
| --- | --- | --- |
| Oral function limitation (ref. comfort) | 1.70 (1.17–2.46) | < 0.01** |
| Sex | 0.78 (0.37–1.65) | 0.52 |
| Age | 1.03 (0.98–1.09) | 0.22 |
| Education duration (years) | 0.94 (0.89–0.99) | < 0.05* |
| Household income | 1.02 (0.85–1.23) | 0.80 |
| Spouse (ref. No) | 0.94 (0.59–1.49) | 0.85 |
| Alcohol experience (ref. No) | 0.86 (0.57–1.30) | 0.48 |
| Smoking (ref. Non-smoker) | 0.80 (0.43–1.49) | 0.48 |
| Sleep duration | 0.96 (0.85–1.08) | 0.50 |
| Physical activity (mild) | 1.03 (0.96–1.10) | 0.43 |
| Nutrition status (MNA-SF score, 0–14) | 0.91 (0.79–1.04) | 0.16 |
| Body mass index | 1.10 (1.02–1.17) | <0.01** |
| Cognitive function (MMSE score, 0–30) | 1.01 (0.96–1.07) | 0.65 |
| Toothbrushing frequency (times/day) | 1.10 (0.86–1.38) | 0.49 |
| Dental check-up (ref. No) | 0.96 (0.64–1.44) | 0.83 |
| Cardiovascular disease (CVD _burden, 0-6) | 1.60 (1.19–2.13) | <0.001*** |
| Diabetes (ref. No) | 0.67 (0.38–1.18) | 0.16 |
| Dyslipidemia (ref. No) | 0.83 (0.54–1.26) | 0.38 |
| Osteoporosis (ref. No) | 2.19 (1.38–3.46) | <0.001*** |
| Thyroid dysfunction (ref. No) | 0.93 (0.44–1.98) | 0.85 |
| Polypharmacy | 1.06 (1.01–1.12) | <0.05* |
| Total cholesterol(mg/dL) | 1.00 (0.99–1.00) | 0.64 |
| Triglycerides (mg/dL) | 1.00 (1.00–1.01) | 0.95 |
| HbA1c (%) | 1.32 (1.05–1.64) | < 0.05* |
| hs-CRP (mg/L) | 0.92 (0.81–1.06) | 0.26 |

**Notes:** SHR (95% CI): Subdistribution Hazard Ratio (95% confidence Interval)

**Abbreviations:** HbA1C, Glycated Hemoglobin; hs-CRP, high-sensitivity C-Reactive Protein.

*P-value: P<0.05*; P<0.01**; P<0.001***

Supplementary Table 4. Description of the eight components comprising the Korean Frailty Index (KFI)

| No. | Component | Scoring Criteria | 0 point | 1 point | Diagnosis Criteria |
| --- | --- | --- | --- | --- | --- |
| 1 | Hospitalization in the past year | Presence of inpatient admission | No | ≥1 time | 0-2: Robust  3-4: Pre-frail  ≥5: Frail |
| 2 | Self-rated health | Subjective perception of health | Good | Poor |  |
| 3 | Polypharmacy | Regular intake of ≥4 prescription drugs | No | Yes |  |
| 4 | Unintentional weight loss | Unintentional weight loss in the past year enough to make clothes loose | No | Yes |  |
| 5 | Depressed mood | Felt sad or depressed for ≥2 weeks during the past year | No | Sometimes or often |  |
| 6 | Incontinence | Difficulty in controlling urination or defecation | No | Sometimes or often |  |
| 7 | Timed Up and Go test | Mobility and balance test result | ≤10 sec | >10 sec |  |
| 8 | Sensory impairment | Difficulty hearing or seeing in daily life | Normal | Impaired |  |

**Note:** Each component is scored as 0 (no deficit) or 1 (deficit), resulting in a total score range of 0–8. The total score was used to classify frailty status in the main analysis.
